# Gastrointestinal Tract Microbiome-Derived Pro-inflammatory Neurotoxins in Alzheimer’s Disease

**Published:** 2021-05-27

**Authors:** Yuhai Zhao, Vivian Jaber, Walter J. Lukiw

**Affiliations:** 1 Department of Anatomy and Cell Biology, Louisiana State University, New Orleans, USA; 2 LSU Neuroscience Center, Louisiana State University Health Science Center, New Orleans, USA; 3 Department of Ophthalmology, LSU Neuroscience Center Louisiana State University Health Science Center, New Orleans, USA; 4 Department Neurology, LSU Neuroscience Center Louisiana State University Health Science Center, New Orleans, USA

**Keywords:** Aging, Bacterial amyloid, *Bacteroides fragilis*, Dysbiosis, Microbiome

## Abstract

The microbiome contained within the human gastrointestinal (GI)-tract constitutes a highly complex, dynamic and interactive internal prokaryotic ecosystem that possesses a staggering diversity, speciation and complexity. This repository of microbes comprises the largest interactive source and highest density of microbes anywhere in nature, collectively constituting the largest ‘diffuse organ system’ in the human body. Through the extracellular fluid (ECF), cerebrospinal fluid (CSF), lymphatic and glymphatic circulation, endocrine, systemic and neurovascular circulation and/or central and peripheral nervous systems (CNS, PNS) microbiome-derived signaling strongly impacts the health, well-being and vitality of the human host. Recent data from the Human Microbiome Initiative (HMI) and the Unified Human Gastrointestinal Genome (UHGG) consortium have classified over ^~^200 thousand diverse, non-redundant prokaryotic genomes in the human GI-tract microbiome involving about ^~^5 thousand different GI-tract microbes that all together encode almost ^~^200 million different protein sequences. While the largest proportion of different microbiome-derived proteins, lipoproteins and nucleic acids provide essential microorganism-specific gene products necessary to support microbial structure, function and viability, many of these same components are also shed from the outer cell wall of different Gram-negative bacterial species into surrounding biofluids which eventually enter the systemic circulation. Several of these microbial-derived secreted molecular species represent some of the most pro-inflammatory and noxious neurotoxins known. These neurotoxins disrupt cell-cell adhesion and easily translocate across aged or damaged plasma membranes and into the systemic circulation, brain, and CNS and PNS compartments. For example, microbial lipoprotein glycoconjugates such as Gram-negative bacteria-derived lipopolysaccharide (LPS), bacterial amyloids and more recently small non-coding RNA (sncRNA) microbial-derived neurotoxins have been found by many independent research groups to reside within the brain cells and CNS tissues of aged patients affected with Alzheimer’s disease (AD). This ‘Commentary’ will highlight the most recent findings on these microbial-derived secreted toxins, their neurotropic properties and the potential contribution of these neurotoxic and pro-inflammatory microbial exudates to age-related inflammatory neurodegeneration, with specific reference to the human GI-tract abundant Gram-negative anaerobe *Bacteroides fragilis* and to AD wherever possible.

## INTRODUCTION

The human gastrointestinal (GI)-tract microbiome forms a highly complex and interactive internal prokaryotic ecosystem that possesses a staggering diversity, speciation and complexity. This dynamic repository of microbes comprises the largest interactive source and highest density of microbes anywhere in nature, collectively constituting the largest ‘diffuse organ system’ anywhere in the human body that is at least as metabolically active as the liver [1–10]. Through the extracellular fluid (ECF), cerebrospinal fluid (CSF), lymphatic and glymphatic circulation, endocrine, systemic and neurovascular circulation and/or central and peripheral nervous systems (CNS, PNS) this microbiome strongly impacts the health, well-being and vitality of the human host [3–9]. Data from the Human Microbiome Initiative (HMI) and the Unified Human Gastrointestinal Genome (UHGG) consortium have recently classified over ^~^200 thousand diverse, non-redundant prokaryotic genomes in the human GI-tract microbiome involving about ^~^5 thousand different GI-tract microbes that all together encode almost ^~^200 million different protein sequences [4,5]. While the largest proportion of different microbiome-derived proteins, lipoproteins and nucleic acids provide essential microorganism-specific gene products necessary to support microbial structure, function and viability, many of these components are also shed from the outer cell wall of different Gram-negative bacterial species into surrounding biofluids and/or the systemic circulation. Several of these microbial-derived species secreted from GI-tract microbes represent some of the most pro-inflammatory and neurotoxic entities known, and these same secreted neurotoxins disrupt cell-cell adhesion and easily translocate across leaky plasma membranes and into the systemic circulation, brain, CNS and PNS. For example, microbial proteins such as Gram-negative bacteria-derived lipopolysaccharide (LPS), bacterial amyloids and more recently small non-coding RNA (sncRNA) microbial-derived neurotoxins have been found by many independent research groups to reside within the brain cells and CNS tissues of patients affected with Alzheimer’s disease (AD). This ‘Commentary’ will highlight the most recent findings on these microbial derived secreted toxins, their neurotropic properties and the potential contribution of these neurotoxic and pro-inflammatory microbial exudates to age-related inflammatory neurodegeneration, with specific reference to the human GI-tract abundant Gram-negative anaerobe *Bacteroides fragilis* and to AD wherever possible. This paper will also highlight and comment upon recent work from peer-reviewed studies that advance the concept of microbiome-derived bacterial factors, such as microbial-secreted neurotoxins, are critical in driving pro-inflammatory degenerative neuropathology such as those widely observed in the aged AD brain and CNS.

## OVERVIEW

### The human microbiome

Common to all higher eukaryotes, *Homo sapiens* contains a highly dynamic and interactive community of microorganisms known as ‘the human microbiome’ consisting mostly of aerobic and anaerobic bacteria, archaebacteria, fungi, protozoa, viruses and other microorganisms. The human microbiome forms a significant fraction of the human ‘metaorganism’ with considerable commensal and/or symbiotic benefit to the human host [6–10]. Both aerobic and anaerobic Gram-positive and Gram-negative bacteria of the gastrointestinal (GI)-tract constitute the largest proportion of the human microbiome by far, and the impact of microbial secretions on human neurological health and disease is becoming increasingly recognized. *Bacteroidetes*, the largest phylum of anaerobic Gram-negative bacteria in deeper regions of the GI-tract microbiome, while generally beneficial to the host when confined to the interior of the GI-tract, have potential to secrete a remarkably complex array of pro-inflammatory neurotoxins that include microbial surface lipopolysaccharide (LPS), highly immunogenic bacterial amyloids, proteolytic peptides, proteinases, lipoproteins and regulatory nucleic acids consisting mostly of small non-coding RNA (sncRNA). The deleterious neurotoxic effects of these bacterial exudates become more significant as GI-tract and blood-brain barriers (BBB) become altered, leaky and/or dysfunctional in their permeability with aging and disease, including primarily, gastrointestinal, systemic vascular and neurovascular disease [11–15].

### *Bacteroides fragilis* and secretory elements of the human GI-tract microbiome

Approximately ^~^99.5% of all of the resident microbes of the human GI-tract microbiome consist of facultative and/or obligate anaerobic bacteria from just 2 of the major bacterial divisions *Firmicutes* and *Bacteroides*; these form the ‘bacterial core’ the human GI-tract microbiome [14–18]. The ^~^3.5 cm diameter ^~^7 m long human GI-tract varies in pH and oxygen availability along its length; microbes in the deeper and more anaerobic regions of the small intestine are the most enriched in obligate anaerobic microbial species [19,20]. In deep GI-tract regions the most abundant Gram-negative bacteria consists mostly of the phylum Bacteriodetes, with a major genus-species being represented by the obligate Gram-negative anaerobe, non-spore forming bacillus *Bacteroides fragilis* [21–23]. The genus *Bacteroidetes* and in particular the species *Bacteroides fragilis*: (i) is among the most studied and genetically understood human GI-tract resident microorganisms [21,23]; (ii) reside and proliferate exclusively in the GI-tract of higher mammals [24,25]; (iii) colonize deep sections of the human GI-tract where microbial densities approach 8 × 1010 CFU/cm^3^, the highest density of any microbial colonization known in nature [26]; (iv) in some deep human GI-tract regions *B. fragilis* are present at about one-hundred times the abundance of Gram-negative bacilli of the phylum *Proteobacteria* and the genus-species *Escherichia coli* [27]; (v) lie at the core of the human GI-tract microbiome in both American and European populations [28–30]; (vi) exhibit a significant amount of intra-species genomic diversity and associated range and variety of potential biochemical functions [31]; (vii) normally constitute an abundant repository of commensal, symbiotic bacteria generally highly beneficial to human immune-, digestive-, nutritive- and neurological health [30–31]; and (viii) on the other hand enterotoxigenic forms of these same microbes can generate some of the most potent pro-inflammatory and pathogenic neurotoxins known [21,27,32]. These neurotoxins induce pro-inflammatory signaling intermediates such as reactive oxygen species (ROS) which in turn up-regulate a series of pro-inflammatory transcription factors such as NF-kB and NF-kB-sensitive transcription to up-regulate pro-inflammatory microRNAs that include miRNA-146a within AD brain and in transgenic AD (TgAD) murine models [12,32,33]. Over the last 4 years it has been increasingly reported by multiple research groups that *B. fragilis* can secrete: (i) a particularly potent, pro-inflammatory and unique LPS subtype (BF-LPS); (ii) bacterial proteolipids, endo/exotoxins and a highly neurotoxic zinc-metalloproteinase known as *B. fragilis*-toxin (BFT) or fragilysin; and (iii) more recently, bacterial-derived miRNA-like sncRNAs now known to serve a number of critical pathological functions. Recent developments in the analysis and characterization of these 3 major classes of *B. fragilis*-derived neurotoxins are briefly reviewed and commented upon below.

### Lipopolysaccharide (LPS) and *Bacteroides fragilis*-derived LPS (BF-LPS)

Probably the most well-characterized of any neurotoxic species secreted by anaerobic Gram-negative bacteria in the human GI-tract are highly immunogenic amphipathic glycoconjugates known as lipopolysaccharide (LPS). Normally LPS protects Gram-negative bacteria from environmental, chemical and physical stress, and is also recognized by the host innate-immune system upon infection inducing potent pathophysiological effects, strong inflammatory signaling and neuronal cell membrane damage. LPS is typically shed from the outer membrane of Gram-negative bacteria and consist of a hydrophobic lipid-A domain attached to a core oligosaccharide (core-OS) and a distal O-antigen (also known as an O-polysaccharide) [6,34–41]. LPS core-OSs often contain highly immunogenic non-carbohydrate components including amino acids, phosphate and/or ethanolamine substituents and are highly diverse in composition amongst bacterial species, and even within strains of the same species [29,34,37,41–45]. Recent studies indicate that LPS generation, abundance and secretion is stimulated: (i) by proliferation of the Gram-negative bacterial source itself; (ii) by stressors including neurotoxic ROS-inducing environmental metallotoxins such as aluminum and mercury [12,33,35]; and (iii) by other inducers, including those in the diet, such high fat-cholesterol (HF-C) consumption and insufficient dietary fiber, and by other unhealthy lifestyle factors [28–31,36–38].

Dietary deficiency-induced neurotoxicity of LPS are mediated in part by macrophages and microglia through the actions of tumor necrosis factor alpha (TNFα), interleukin-1β (IL-1β) and reactive oxygen species (ROS), which may be three of the most important endogenous mediators of the toxic pathophysiological effects of LPS [38–41]. Each Gram-negative bacillus, indeed each GI-tract resident microbe has the potential to secrete a slightly different LPS and/or neurotoxin assortment with slightly different lipid and oligosaccharide domain structures, abundances, activities, molecular properties and toxicities [41–47]. The modulatory effect of dietary fiber on the propagation of these microbial species and their neurotoxins remain incompletely understood but is a very active field of research investigation as different kinds and amounts of dietary fiber are now known to influence the proliferation, and hence neurotoxin-promoting activities of *B. fragilis* [21–23,42,48–50]. Interestingly, different fiber types such as soluble versus insoluble dietary fiber, appears to have different effects on the propagation and growth of these enterotoxigenic microbes in the human GI-tract microbiome and their microbe-derived neurotoxin-mediated effects, as well as on the proliferation and maintenance of microbial diversity [31,39,45–48].

LPS can be therefore considered as a leading example of a highly immunogenic, Gram-negative bacterial-derived amphipathic neurotoxic glycoconjugate, and it has been recently convincingly shown by multiple research laboratories that a diet-inducible BF-LPS accumulates in AD-affected neurons to ultimately deregulate homeostatic gene expression patterns [6,29,45,48–52]. Interestingly LPS has a particularly high affinity for human neocortical neuronal plasma membranes, and this attraction is significantly enhanced in the presence of the amyloid-beta 42 (*Aβ42*) peptides that accumulate in AD-affected brain [29,45].

### Bacterial amyloid-neurotoxic proteolytic peptides, metalloproteinase and lipoproteins

Bacteria thrive in dense multicellular communities held together by an extracellular matrix, and the major proteolipid component of this matrix are highly stable and structured bacterial amyloids and lipoproteins [52,53]. While secreted LPS, bacterial amyloids and polypeptides are generally quite soluble as monomers over time they coalesce into highly insoluble pro-inflammatory fibrous protein aggregates during the course of aging that are implicated in progressive degenerative neuropathology, neurotoxicity and immunomodulation in several common, age-related disorders of the human CNS including Parkinson’s disease (PD), prion disease (PrD) and AD. Ongoing studies indicate that in concert with BF-LPS and/or BFT, bacterial amyloids aid in the disruption of paracellular- and transcellular-barriers by cleavage of intercellular-proteins resulting in ‘leaky’ and compromised barriers [21–25,53]. These barriers: (i) become defective and more penetrable with aging and disease; and (ii) permit entry of microbiome-derived neurotoxins into the systemic-circulation from which they next transit the blood-brain barrier and gain access to the brain and CNS [21–23,53,54]. Here LPS and bacterial amyloids accumulate, induce neuroinflammation and significantly alter homeostatic patterns of brain gene expression. Other potentially neurotoxic GI-tract-derived components of the microbiome recently described, besides LPS, bacterial amyloid and proteolipids include partially catabolized bacterial amyloid polypeptide fragments and the metabolites of bacteria including short-chain fatty acids (SCFAs), branched amino acids and neurotransmitters, and functional by-products such as steroid carboxylic acids derived from cholesterol including cholic, chenodeoxycholic acids and other related forms of bile acids [6,51–53].

### GI-tract microbial-derived small non-coding RNA (sncRNA)

Very recently microbiome-derived microRNA-like sncRNAs have been shown to be directly involved in the support of multiple pathogenic AD-relevant processes in both human brain and CNS tissues and *in vitro* studies of human neural cells in primary culture. For example human miRNA-like sncRNAs have been recently demonstrated: (i) to contribute to the virulence of bacterial outer membrane proteins [55]; (ii) to regulate the adaptation of Gram-negative GI-tract bacteria to the complex prokaryotic environment of the GI-tract [56]; (iii) as microRNAs are induced *via* a common LPS-ROS-NF-kB-microRNA signaling pathway relevant to pathological gene expression changes as are observed in AD [12,14,15,49]; (iv) to act as transcriptional riboswitches that regulate bacterial transcription elongation and termination, thus turning downstream gene expression “on” or “off and regulating critical gene expression control across time [57]; and (v) to be involved in controlling and enhancing CRISPR-CAS systems both within bacteria and affecting host cells [58]; Interestingly sncRNAs and microRNAs are found to be an abundant ribonucleic acid components of complex molecular cargos in communicating vesicles that are capable of translocation and communication between the aged GI-tract microbiome and AD-affected neocortical brain cells and tissues [6,12,59,60].

## DISCUSSION

Recent and ongoing research is significantly expanding our understanding of the role of GI-tract microbiome-derived secreted pro-inflammatory neurotoxic exudates in PD, PrD and AD and strengthening the idea that GI-tract microorganisms and/or their secretory components contribute to progressive, age-related inflammatory neurodegeneration. This enormous repository of GI-tract microbiome-derived neurotoxins are derived from over ^~^200 thousand diverse, non-redundant prokaryotic genomes that generate ^~^200 million proteins [5,61,62]. While the majority of these different proteins/lipoproteins provide critical components of microbial structure, function and *via*bility, some of these components are also beginning to be observed to be shed from the outer cell wall of Gram negative bacteria, and also from other microbial species, into the surrounding biofluids that eventually find their way across the GI-tract and blood-brain barriers (BBB) into the brain and CNS. These secreted biopolymers are extremely pro-inflammatory and highly toxic to neuronal structure and function and the homeostatic signaling functions of human neural cells. Other aspects related to GI-tract microbiome complexity and diversity are that: (i) this large microbial repository altogether actively generate extremely pathogenic pro-inflammatory and pathogenic neurochemical cocktails detrimental to the overall function and survival of neurons and CNS and/or PNS tissues; and (iii) while secreted LPS, amyloids, other lipoproteins and sncRNA are generally quite soluble as monomers over time they self-associate into highly insoluble fibrous protein aggregates that appear within and around neurons and throughout the neocortex and brain parenchyma. These have long been implicated in the progressive degenerative neuropathology of several progressive, age-related disorders of the human CNS including PD, PrD and AD. The evolving data-driven concept that each human individual possesses an individualistic GI-tract microbiome that varies in microbial abundance, complexity and speciation may predispose individual humans to differential susceptibility to different forms of age-related neurological disease [5,61–63].

## CONCLUSION

This further implies that each human possesses a unique metagenomic microbial profile which may be exploitable for microbiotic augmentation, personalized medicine and/or the implementation of individually-directed therapeutic strategies useful in the clinical management of inflammatory neurodegenerative disorders such as AD and other types of age-related neurological disease.

## ABBREVIATIONS:

AD: Alzheimer’s disease
LPS: lipopolysaccharide
miRNA-146a: microRNA-146a
ROS: reactive oxygen species
